# Visualizing tumor evolution with the fishplot package for R

**DOI:** 10.1186/s12864-016-3195-z

**Published:** 2016-11-07

**Authors:** Christopher A. Miller, Joshua McMichael, Ha X. Dang, Christopher A. Maher, Li Ding, Timothy J. Ley, Elaine R. Mardis, Richard K. Wilson

**Affiliations:** 1 McDonnell Genome Institute, Washington University School of Medicine, St. Louis, MO 63108 USA; 2 Department of Medicine, Division of Genomics and Bioinformatics, Washington University School of Medicine, St. Louis, MO 63108 USA; 3 Department of Medicine, Division of Oncology, Washington University School of Medicine, St. Louis, MO 63108 USA; 4 Department of Genetics, Washington University School of Medicine, St. Louis, MO 63108 USA

## Abstract

**Background:**

Massively-parallel sequencing at depth is now enabling tumor heterogeneity and evolution to be characterized in unprecedented detail. Tracking these changes in clonal architecture often provides insight into therapeutic response and resistance. In complex cases involving multiple timepoints, standard visualizations, such as scatterplots, can be difficult to interpret. Current data visualization methods are also typically manual and laborious, and often only approximate subclonal fractions.

**Results:**

We have developed an R package that accurately and intuitively displays changes in clonal structure over time. It requires simple input data and produces illustrative and easy-to-interpret graphs suitable for diagnosis, presentation, and publication.

**Conclusions:**

The simplicity, power, and flexibility of this tool make it valuable for visualizing tumor evolution, and it has potential utility in both research and clinical settings. The fishplot package is available at https://github.com/chrisamiller/fishplot.

**Electronic supplementary material:**

The online version of this article (doi:10.1186/s12864-016-3195-z) contains supplementary material, which is available to authorized users.

## Background

Most cancers are heterogeneous and contain multiple subclonal populations that can be detected via high depth massively parallel sequencing. An increasing number of studies are collecting and sequencing longitudinal samples, allowing the clonal evolution of tumors to be tracked in detail. Though tools have been developed for inferring subclonal architecture [1–4] and for determining tumor phylogeny [5, 6], few offer compelling and intuitive visualizations.

We reported one of the first studies describing tumor evolution defined by whole genome sequencing, in patients with relapsed Acute Myeloid Leukemia (AML), and that publication contained a series of custom figures showing changes in clonal architecture between the primary and relapse presentations [7]. Often called “fish plots” due to their resemblance to tropical fish, these visualizations have become widely adopted, both by our group and others [8–11]. Until now, each has been created in vector-art programs like Adobe Illustrator, which is laborious and makes representing accurate proportions challenging. As the sizes of cohorts have grown, this approach has quickly become untenable.

To enable the creation of these plots in a robust and automatable fashion, we have developed an R package (“fishplot”) that takes estimates of subclonal prevalence at different timepoints, and outputs publication-ready images that accurately represent subclonal relationships and their relative proportions. Fishplot is available at https://github.com/chrisamiller/fishplot.

## Implementation

The fishplot package was implemented in R, and requires a minimal set of dependencies (the “plotrix”, “png”, and “Hmisc” packages). Several inputs, including the clonal fractions of each tumor cell population, a representation of descent in the form of parental relationships, and the timepoints at which the samples were obtained are required. These data are readily available from existing tools, such as the clonevol package, which already includes code that interfaces with fishplot for visualization (https://github.com/hdng/clonevol). This feature enables seamless integration into existing genomic analysis pipelines. Figures are output through the R standard graphics libraries, which allow for the creation of vector or raster-based images of any size, suitable for a wide range of applications.

## Results

**Fig. 1:**
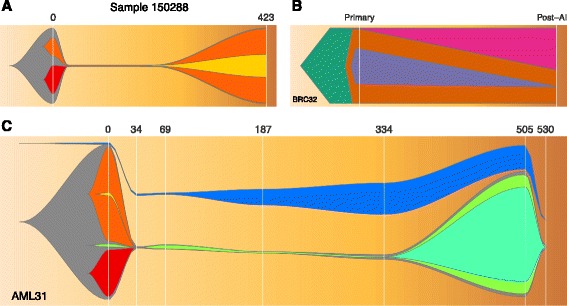
Visualizing tumor evolution with the fishplot package. Panel **a** A case of primary and relapsed AML. Panel **b** A breast cancer before and after neoadjuvant aromatase inhibitor therapy. Panel **c** An AML with complex clonal structure and 7 timepoints

We have applied fishplot to a number of different cancer genomics studies, and three representative results are displayed in Fig. 1.

We first created a fish plot of an AML patient with a chemotherapy-induced bottleneck that eliminated one subclone, while another survived and drove the relapse (Fig. 1a). This plot uses the default color scheme and the default curve splining method for smoothing, along with timepoint labels representing the number of days since tumor presentation.

We next plotted a breast tumor sequenced before and after 4 months of neoadjuvant aromatase inhibitor therapy [12] (Fig. 1b). This therapy did not induce an extreme bottleneck, but nonetheless resulted in substantial clonal remodeling. The resulting figure uses user-defined colors and represents subclones as polygons, without curve smoothing. The standard numeric labels were replaced with categorical labels, but the timepoints remain scaled appropriately.

Lastly, we created a model of AML31, a patient that was sampled with ultra-deep sequencing at many timepoints, allowing even very rare (<1 % Variant Allele Frequency) subclones to be detected [9] (Fig. 1c). Chemotherapy did not completely eliminate the cancer in this case, resulting in detectable levels of tumor until relapse at day 505 (with subsequent clearance of the tumor with via salvage chemotherapy). The fishplot shows this progression, including the failure to completely clear the tumor. This patient also had oligoclonal skewing post-chemotherapy, resulting in clonal expansion from a hematopoetic stem cell that was not part of the patient's leukemia (Fig. 1c, blue). The package includes functionality for representing such unrelated clones, which is also useful in the case of “collision tumors” with independent origins.

The code and data used to produce Fig. 1 is available as Additional file 1 and can be also found within the example scripts in https://github.com/chrisamiller/fishplot/blob/master/tests/test.R Additional file 2 contains an analysis pipeline demonstration that chains together the sciClone, clonevol, and fishplot packages, taking data from raw somatic variant calls through subclonal detection, phylogeny inference, and fishplot creation.

## Conclusions

Characterizing subclonal architecture and the ways in which tumors evolve, both over time and in the context of therapeutic intervention, is important for understanding therapy resistance, which contributes to tens of thousands of cancer deaths each year. Fish plots, like those presented here, provide researchers and clinicians with an intuitive and accurate representation of how an individual tumor is changing over time, potentially making analysis and diagnosis easier. Despite being designed for tracking tumor evolution, this tool may also find niches outside of cancer biology, and could easily be used to represent the changing landscapes of microbial populations, for example. Our group has already used images created by the fishplot package in a number of genomic pipelines and pending publications, and we anticipate that it will be adopted widely within the large community of scientists studying tumor evolution.

## Availability and requirements

The fishplot package has been tested on R versions > 2.15 and requires the “plotrix”, “Hmisc”, and “png” packages. It is available from https://github.com/chrisamiller/fishplot.

## Additional files

Additional file 1: Figure 1 example: The code and data used to produce Fig. 1. (R 2 kb)
Additional file 2: Analysis Pipeline example: an example that uses the sciClone, clonevol, and fishplot packages to take data from raw somatic variant calls through subclonal detection, phylogeny inference, and fishplot creation. (ZIP 29 kb)

## Abbreviations

AML: Acute Myeloid Leukemia
